# Effects of Acute Abdomen III on Sepsis-Induced Intestinal Damage in a Rat Model

**DOI:** 10.4014/jmb.2412.12067

**Published:** 2025-06-26

**Authors:** Lu Wang, Jingjing Hu, Fangyu Hu, Yuexuan Chen, Ming Fang, Yuanling Ye

**Affiliations:** 1 Department of Emergency, Hangzhou TCM Hospital Affiliated to Zhejiang Chinese Medical University, P.R. China; 2 Department of Respiratory, Hangzhou TCM Hospital Affiliated to Zhejiang Chinese Medical University, P.R. China

**Keywords:** Sepsis, intestinal barrier, acute abdomen III, oxidative stress, inflammation

## Abstract

The disruptions to intestinal integrity contribute to sepsis-related complications. Acute abdomen III is a traditional Chinese medicine formula. The objective of this research is to investigate the impact of acute abdomen III upon sepsis-induced intestinal damage. A rat model of cecal ligation and puncture was used to evaluate the impact of acute abdomen III on sepsis-induced intestinal damage. Histopathological analysis of intestinal tissue damage, detection of systemic inflammation and measurement of tight junction protein were performed by hematoxylin and eosin staining, enzyme-linked immunosorbent assay, and Western blot, respectively. Oxidative stress and intestinal barrier integrity were assessed. Terminal deoxynucleotidyl transferase dUTP nick end labeling was employed to evaluate apoptosis. Expression levels of the apoptosis-related proteins were examined. Model rats treated with acute abdomen III exhibited significantly mitigated intestinal damage. Acute abdomen III treatment reduced systemic inflammation and oxidative stress, as evidenced by downregulation of malondialdehyde and upregulation of superoxide dismutase and catalase. Acute abdomen III therapy lowered endotoxin, D-lactate, and diamine oxidase levels, while boosting the levels of tight junction proteins ZO-1, claudin-1 and occludin, implying an enhancement in intestinal barrier integrity. Acute abdomen III also markedly suppressed apoptosis in intestinal epithelial cells. Acute abdomen III can protect against sepsis-induced intestinal damage by reducing systemic inflammation and oxidative stress while promoting intestinal barrier integrity.

## Introduction

Organ malfunction, as a consequence of dysregulated host responses to infection, is a hallmark of sepsis (a potentially fatal illness) [1], with incidence on the rise and an estimated 49 million cases reported globally each year [2]. Sepsis remains one of the lethal factors in hospitalized patients, the mortality rate of which varies from 20% to 60%, depending on severity and comorbidities [3]. It is widely accepted that the initial high inflammatory response to sepsis causes tissue damage, leading to organ failure [4]. The typical immune responses of sepsis involve an exaggerated pro-inflammatory reaction to pathogen epitopes, alongside simultaneous activation of anti-inflammatory pathways [5], various mechanisms of which have been widely discussed. Shi *et al*. reported that ASGR1 modulates the levels of interleukin-1 beta (IL-1β), IL-6, and tumor necrosis factor-alpha (TNF-α) through the nuclear factor-kappa B/activating transcription factor 5 (NF-κB/ATF5) pathway, resulting in sepsis-induced liver damage [6]. Janicova *et al*. pointed out that endogenous uteroglobin controls cytotoxicity, viability, and monocyte-mediated release of transforming growth factor beta-1 (TGF-β1) that induces lung injury in sepsis [7]. Zou *et al*. found that sepsis increases plasma levels of microRNA-146a-5p, which exacerbates blood-brain barrier (BBB) disruption via a toll-like receptor 7 (TLR7)-dependent mechanism, and thus aggravates brain inflammation [8]. Current treatments for sepsis resort to early and aggressive antibiotic therapy, fluid resuscitation, and organ support in intensive care settings [9]. Despite broad research, the prognosis for severe sepsis remains grim, underscoring the necessity for developing novel therapeutic approaches and identifying reliable prognostic biomarkers.

Traditional Chinese medicine (TCM) is an ancient healthcare system with thousands of years of history, comprising herbal medicine, acupuncture, and other treatments designed to promote balance and health [10, 11]. TCM is recognized globally for its potential therapeutic benefits. For instance, ginsenoside Rh2 (G-Rh2) exhibits anticancer activity by inhibiting the Axl signaling pathway and reducing colorectal cancer cell growth [12]. Astragalus polysaccharides help prevent bone loss in postmenopausal women via dwindling osteocalcin (OCN) and TNF-α protein levels [13]. One specific TCM formula, acute abdomen III, consists of *raw rhubarb*, *magnolia officinalis*, *aurantii fructus*, *salvia miltiorrhiza*, *taraxaci herba*, and *sargentodoxa cuneata*. Clinically, acute abdomen III has been used to mitigate gastrointestinal dysfunction caused by sepsis and reduce the severity of sepsis [14]; however, the exact mechanisms remain unclear.

The present study aims to examine the impact of acute abdomen III upon the systemic release of cytokines in a sepsis model, as well as upon oxidative stress levels in intestinal cells. Additionally, we probed into the role of acute abdomen III in controlling tight junction proteins and intestinal cell apoptosis in sepsis.

## Materials and Methods

### Ethics Statement

Forty male Sprague-Dawley (SD) rats (250-300 g, 8-10 weeks old) were obtained from Hangzhou Medical College. The rats were kept under controlled conditions with a temperature of approximately 20°C, relative humidity of about 45% and a 12-h light/dark cycle. They were fed with conventional lab food and given tap water. All operations obtained approval from Ethics Committee of Zhejiang Baiyue Biotech Co., Ltd. for Experimental Animals Welfare (No.ZJBYLA-IACUC-20230721) and followed the rules on the control of experimental animals published by the Ministry of Health of China.

### Cecal Ligation and Puncture (CLP) Procedure

During the surgical procedure, a heating pad was used to keep the rats’ core body temperature between 36°C and 38°C. Anesthesia was induced with 4% isoflurane (26675-46-7, MilliporeSigma, USA). A midline incision was made to carefully isolate and ligate the cecum below the ileocecal valve. After two punctures to the cecum with a 20-gauge needle, the abdominal cavity was sealed with 2 layers of sutures [15]. Then, the rats received subcutaneous fluid resuscitation. Throughout the surgery, great care was taken to avoid damage to blood vessels.

### Drug Administration

Acute abdomen III is a proprietary formula (500 ml/bottle, 220119) produced by Hangzhou Hospital of Traditional Chinese Medicine. As per the research’s methodology, four groups of SD rats were arbitrarily assigned into sham, model, acute abdomen III-Low (acute abdomen III-L; 10 ml/day), and acute abdomen III-High (acute abdomen III-H; 30 ml/day) groups, with 10 rats in each group. Prior to the CLP procedure, rats in the acute abdomen III groups received pre-treatment via gavage with acute abdomen III for 3 days [14]. After the CLP procedure, rats in the acute abdomen III groups experienced gavage for one more day. The same volume of regular saline was given to rats in the sham and model groups. The rats were sacrificed 2 days after CLP procedures, and samples of intestinal tissue and blood were harvested for additional examination.

### Histopathological Examination and Injury Index

Intestinal tissues were preserved for more than 24 h at 4°C using 4% paraformaldehyde, subsequently embedded in paraffin and cut into 5 μm sections. Hematoxylin and eosin (H&E) staining (C0105M, Beyotime, China) was performed at room temperature, followed by observation with a microscope (DP Olympus BX51, Japan) at 100× magnification. Intestinal damage was assessed using the Chiu scoring system [16]. Three senior pathology professors, blinded to the study content, randomly selected five fields from each tissue section for scoring, and the final score was the average of these scores.

### Enzyme-Linked Immunosorbent Assay (ELISA)

Serum samples were obtained by centrifuging blood samples at 3,000 ×*g* for 15 min at 4°C, in which TNF-α, IL-6, IL-1β, and diamine oxidase (DAO) levels were measured using ELISA kits (E-HSEL-R0001; E-EL-R0015; E-EL-R0012; E-EL-R3013; Elabscience Biotechnology Inc., China). ELISA measurements were performed in microplate reader (Bio-Rad, USA) at 450 nm.

### Endotoxin and D-Lactate Assessment

Serum samples were obtained by centrifuging blood at 3,000 ×*g* for 15 min at 4°C. Endotoxin and D-lactate levels were measured with endotoxin test kits (G2250, EsEN Biotechnology Co., Ltd., China) and D-lactate test kits (K-DATE, Shanghai Xinrui Biotechnology Co., Ltd., China) according to manufacturers’ protocols.

### Oxidative Stress Level Detection

The levels of oxidative stress markers were measured using kits, including superoxide dismutase (SOD), catalase (CAT) and malondialdehyde (MDA). With a bath of ice and the Ultra Turrax IKA T18 Basic homogenizer (IKA Labortechnic, Germany), intestinal tissues were mixed together in phosphate-buffered saline (PBS, pH level = 7.4, D8537, Sigma-Aldrich, USA). The homogenate was spun (14,000 rpm) for 10 min at 4°C to extract the final product. SOD assay kit (S0101S, Beyotime, China) was used to determine SOD levels in accordance with the directions.

A catalase assay kit (70702, Cayman Chemical, USA) was used to measure the concentration of CAT. The enzyme interacted with methanol when hydrogen peroxide (H_2_O_2_) was at right concentrations. CAT activity was determined by quantifying formaldehyde generated through colorimetry.

MDA level was detected as follows. Tissues from the intestine were mixed together in 1.15% potassium chloride (KCl) solution. A mixture that included homogenate (100 μl), 8.1% sodium lauryl sulfate (200 μl), 20% acetic acid (pH level = 3.5, 1,500 μl), 0.8% thiobarbituric acid (1,500 μl), and distilled water (700 μl) was prepared. After 1 h of heating at 95°C, the mixture was spun at 3,000 ×*g* for 10 min. The absorbance of the supernatant at a wavelength of 650 nm was measured with a spectrophotometer.

### Western Blot Analysis

Protein content was measured by radioimmunoprecipitation assay (RIPA) buffer (D1010; Beijing Solarbio Science & Technology Co., Ltd., China). 10 mg tissue samples were rest in 100 μl of buffer in proportion. The resultant cloudy solution was centrifuged for 20 min at 12,000 ×*g* (4°C). The protein content was then measured via a bicinchoninic acid (BCA) assay. Using sodium dodecyl sulfate-polyacrylamide gel electrophoresis, equal amounts of protein (30 μg/lane) were separated and then electroblotted onto a PVDF membrane. The membrane was sealed with 5% non-fat milk for 2 h, and probed with primary antibodies overnight at 4°C, including ZO-1 (ab276131; Abcam, UK), Claudin-1 (ab307692; Abcam), occludin (ab224526; Abcam), Bcl-2 (ab194583; Abcam), Bax (ab32503; Abcam), cleaved Caspase-3 (9664; Cell Signaling Technology, USA) and GAPDH (ab8245; Abcam). Then, incubation with horseradish peroxidase-conjugated Goat Anti-rabbit secondary antibody (ab205718; Abcam) was conducted at 37°C for 90 min. The proteins were then quantified using a chemiluminescence system (ChemiDoc Touch; Bio-Rad Laboratories, Inc., USA) and assessed with ImageLab 5.2 software (Bio-Rad Laboratories, Inc.).

### Intestinal Epithelial Cell Apoptosis

5 μm thick sections were cut from intestinal tissues that had been fixed for two days at room temperature in 4%paraformaldehyde (P6148, Sigma-Aldrich, USA) and encased in paraffin (69018961, Shanghai National Pharmaceutical Chemical Reagents Co., Ltd., China). Following the manufacturer’s guidance, apoptosis was identified using the Terminal deoxynucleotidyl transferase dUTP nick end labeling (TUNEL) test kit (C1086, Beyotime). After deparaffinization, the tissue was hydrated with 0.2% Triton X-100 (9002931; Sigma-Aldrich, USA) to increase cell permeability. The TUNEL reaction solution, the converter-peroxidase, and 3,3'-diaminobenzidine (DAB; D8001, Sigma-Aldrich) were applied to the sections. A drop of DAB substrate (100 μl) was added to each tissue sample for visualization, and color development was stopped upon adequate yellow-brown staining. Next, the slides were covered with neutral balsam and examined with a light microscope (100× magnification). Three professional pathology professors randomly chose five fields from every tissue section and examined them.

### Immunohistochemistry

After being fixed for 48 h at room temperature with 4% paraformaldehyde, intestinal tissues were covered in paraffin, sectioned, incubated in xylene at room temperature for 10 min, and soaked in a series of ethanol solutions (100%, 95%, 85%, 75%, and 50%) and distilled water for 5 min each. Three rounds of washing were performed on the sections using PBS. Next, sections were incubated with 3% hydrogen peroxide (7722-84-1; Sigma-Aldrich) in a humid chamber for 10 min to block endogenous peroxidase activity, followed by washing with PBS to remove the hydrogen peroxide. To obtain antigens, the sections were placed in a citrate buffer (P0083; Beyotime Biotechnology Research Institute, China) in a stainless steel container for 20 min. After being blocked with 10% goat serum (ZLI-9022, ZSGB-BIO, China) in a humid chamber for 30-60 min, the sections were treated at 4°C for a whole night with primary antibodies, Bcl-2 (ab182858; Abcam), Bax (ab32503; Abcam), and cleaved Caspase-3 (#9661; Cell Signaling Technology). The PBS-washed sections were incubated with biotinylated goat anti-rabbit secondary antibody (ab6721, Abcam) at 37°C for 30 min, treated with SABC, DAB and hematoxylin for visualization, and quickly immersed in 1% hydrochloric acid-ethanol and a series of ethanol solutions for dehydration. After sealing with neutral balsam, the sections were examined and photographed.

### Reverse Transcription Quantitative Polymerase Chain Reaction (RT-qPCR)

Total RNA was isolated from intestinal tissue with Trizol reagent (R0016, Beyotime), and its purity and concentration were measured with a UV spectrophotometer (UV1901, Shanghai AoXi Scientific Instruments Co., Ltd., China). The extracted RNA was adjusted to a concentration of 50 ng/μl. RNA was reverse-transcribed with PrimeScript RT Kit (RR047A, Takara Holdings Inc.), and qPCR was performed using a real-time PCR instrument (ABI 7900, Shanghai Pudixin Biological Technology Co., Ltd., China). The reference gene used was glyceraldehyde-3-phosphate dehydrogenase (*GAPDH*). The mRNA expression levels of *Bcl-2*, *Bax*, and *Caspase-3* were analyzed utilizing the 2^-ΔΔCt^ method. The following primer sequences were employed:

*Bcl-2*: Forward 5'-TGTGATGGACTGACTACCTGAACC-3', Reverse 5'-CAGCCAGGAGAAATCAAACAGAGG-3'.

*Bax*: Forward 5'-CGGCGAATTGGAGATGAACTGG-3', Reverse 5'-CTAGCAAAGTAGAAGGGCAACC-3'.

*Caspase-3*: Forward 5'-GTGAGACTGACGATGATATGGC-3', Reverse 5'-CGCAAAGTGACTGGATGAACC-3'.

*GAPDH*: Forward 5'-AACGGATTTGGTCGTATTGG-3', Reverse 5'-TTGATTTTGGAGGGATCTCG-3'.

### Statistical Analysis

To perform the statistical analysis, GraphPad Prism 8.0 was used. Mean ± standard deviation (SD) was used to express the data. Multi-group comparisons were conducted by one-way analysis of variance (ANOVA), followed by Tukey’s test for post hoc comparisons. A *p*-value below 0.05 was applied to determine the significance of the data.

## Results

### Acute Abdomen III Reduced Sepsis-Induced Intestinal Damage

**Fig. 1:**
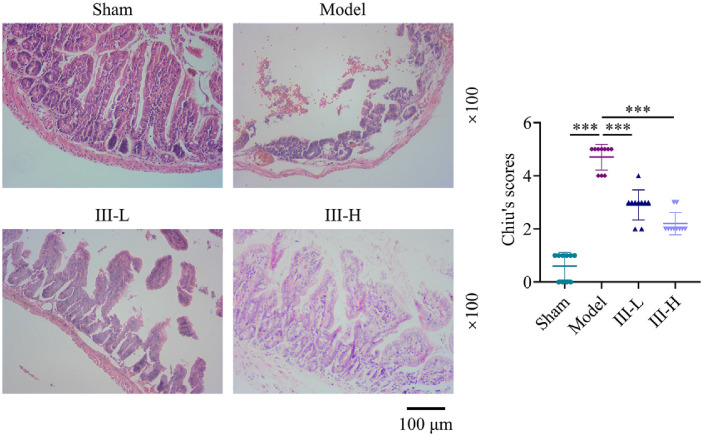
Effect of acute abdomen III on sepsis-induced intestinal damage in rats. Histopathological analysis of intestinal tissue from sepsis-induced rats treated with or without acute abdomen III was performed using hematoxylin and eosin (H&E) staining. Magnification: 100×. The severity of intestinal damage was assessed using the Chiu scoring system in rats treated with or without acute abdomen III. Each group consisted of 10 rats. ***P < 0.001.

To evaluate the impact of acute abdomen III on intestinal tissue damage in rats subjected to CLP-induced sepsis, histopathological analysis with H&E staining was carried out. When compared to the sham group, the model group showed severe intestinal damage (Fig. 1). Rats treated with acute abdomen III exhibited significantly mitigated intestinal damage compared with the model rats, and high-dose acute abdomen III treatment yielded better outcomes than the low-dose treatment (Fig. 1). The Chiu scoring method was used to determine the degree of intestinal damage. The scores for the model group were significantly higher than those for the sham group (*P* < 0.001, Fig. 1). Relative to the model group, the acute abdomen III groups had significantly lower Chiu scores (*P* < 0.001, Fig. 1).

### Acute Abdomen III Mitigated Sepsis-Induced Inflammation, Oxidative Stress, and Intestinal Permeability

**Fig. 2:**
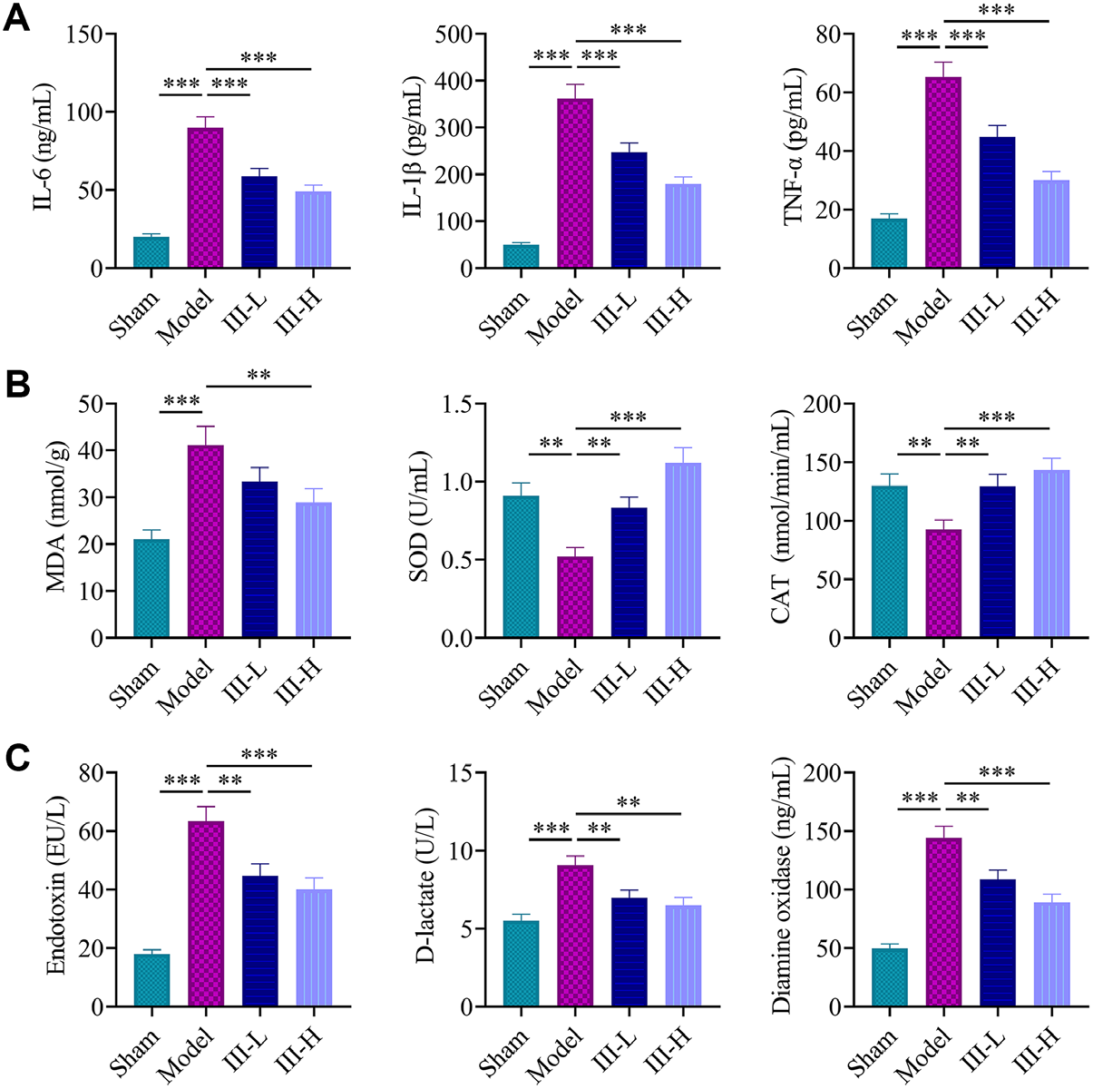
Regulation of inflammation, oxidative damage, and intestinal permeability by acute abdomen III in sepsis-induced rats. Levels of serum interleukin-6 (IL-6), interleukin-1 beta (IL-1β), and tumor necrosis factor-alpha (TNF-α) in sepsis-induced rats treated with or without acute abdomen III were measured using enzyme-linked immunosorbent assays (ELISA). (**B**) Malondialdehyde (MDA), superoxide dismutase (SOD) and catalase (CAT) content in intestinal tissue was determined using spectrophotometry, and corresponding detection kits. (**C**) Endotoxin levels in serum, D-lactate levels, and diamine oxidase (DAO) levels were evaluated using specific test kits in sepsis-induced rats treated with or without acute abdomen III. **P < 0.01; ***P < 0.001. Each group consisted of 3 rats.

ELISA was used to measure systemic inflammatory responses in sepsis model rats by determining concentrations of serum IL-6, IL-1β and TNF-α. The results showed that the model group had considerably greater levels of TNF-α, IL-1β, and IL-6 than the sham group (*P* < 0.001, Fig. 2A). Serum IL-6, IL-1β, and TNF-α levels were lower in acute abdomen III groups than model group (*P* < 0.001, Fig. 2A). Additionally, in the intestinal tissue, the concentrations of MDA, SOD, and CAT were measured. SOD and CAT levels were lower and MDA levels were higher in the model group than in the sham group (*P* < 0.01, Fig. 2B). Relative to the model group, the acute abdomen III groups displayed reduced MDA levels and significantly increased SOD and CAT levels (*P* < 0.01, Fig. 2B). To evaluate intestinal barrier integrity, endotoxin, D-lactate and DAO levels were assessed. The model group had higher levels of endotoxin, D-lactate, and DAO compared to the sham group (*P* < 0.001, Fig. 2C). When comparing acute abdomen III groups with model group, the therapy was confirmed to dramatically decrease the levels of endotoxin, D-lactate, and DAO (*P* < 0.01, Fig. 2C).

### Acute Abdomen III inhibited Sepsis-Induced Intestinal Barrier Damage and Cell Apoptosis

**Fig. 3:**
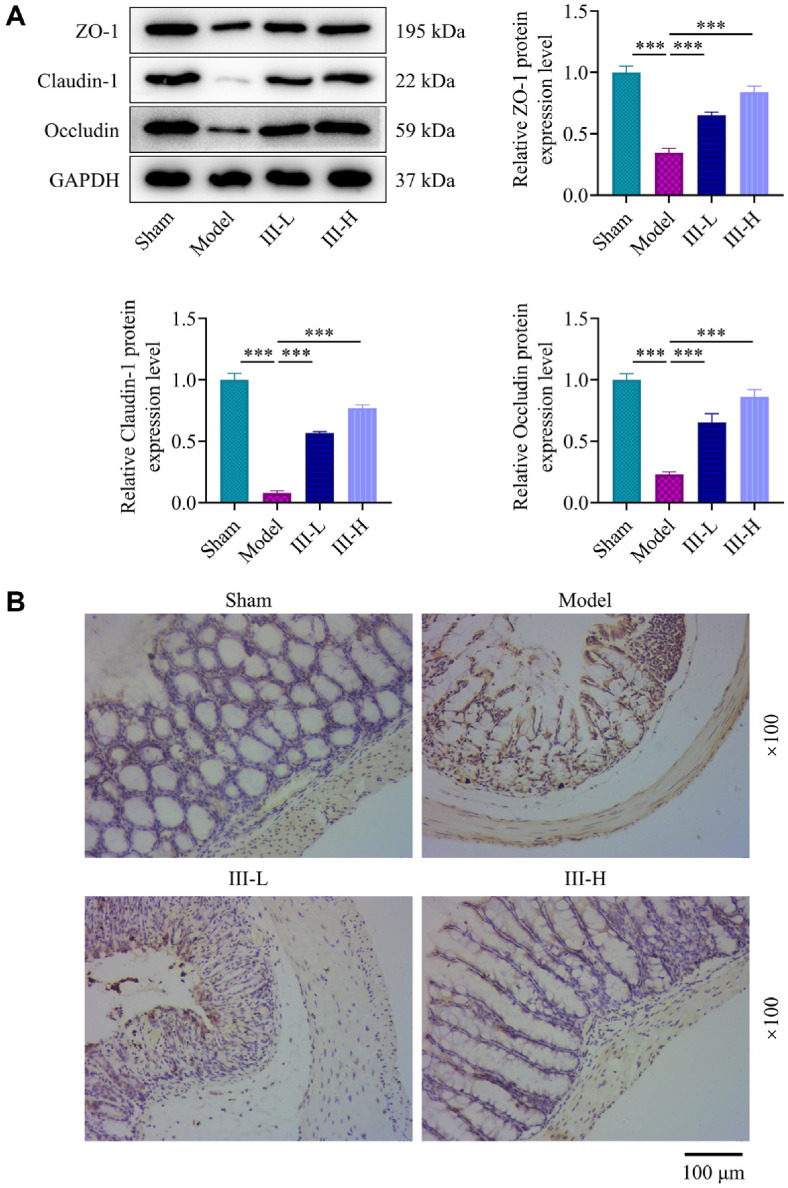
Regulation of intestinal mechanical barrier repair and apoptosis by acute abdomen III in sepsisinduced rats. (**A**) Western blot analysis of ZO-1, claudin-1, and occludin protein levels in intestinal tissue from sepsisinduced rats treated with or without acute abdomen III. GAPDH was used as a loading control. (**B**) Terminal deoxynucleotidyl transferase dUTP nick end labeling (TUNEL) assay was applied to evaluate apoptosis in intestinal tissue in sepsis-induced rats treated with or without acute abdomen III. Magnification: 100×. ***P < 0.001. Each group consisted of 3 rats.

To further assess the role of acute abdomen III in regulating intestinal mechanical barrier integrity, the tight junction protein expressions in intestinal tissues were quantitated using Western blot analysis. The data revealed evident downregulation of ZO-1, claudin-1 and occludin in the model group in contrast with the sham group (*P* < 0.001, Fig. 3A). Relative to the model group, the acute abdomen III groups exhibited a substantial increase in tight junction protein expressions (*P* < 0.001, Fig. 3A). Intestinal cell apoptosis was examined using the TUNEL assay. As shown in Fig. 3B, a small number of apoptotic cells (brown-stained cells) were detected in the sham group, and the number was increased in the model group. Acute abdomen III treatment apparently suppressed sepsis-induced intestinal epithelial cell apoptosis.

### Acute Abdomen III Regulated Expressions of Apoptosis-Related Genes

**Fig. 4:**
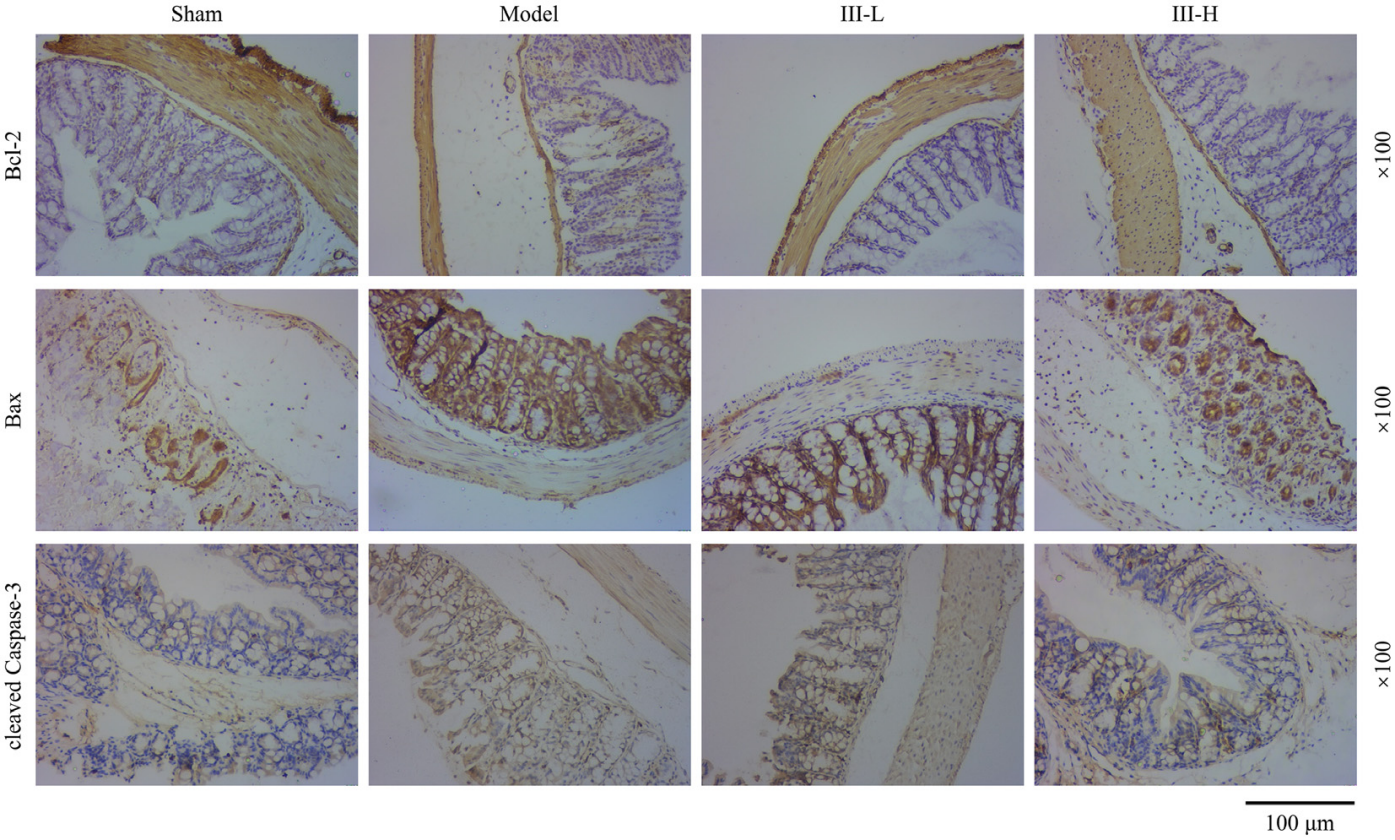
Effect of acute abdomen III on apoptosis markers in intestinal tissue of sepsis-induced rats. Immunohistochemistry analysis of Bcl-2, Bax, and cleaved Caspase-3 levels in intestinal tissue from sepsis-induced rats treated with or without acute abdomen III. Magnification: 100×. Each group consisted of 3 rats.

**Fig. 5:**
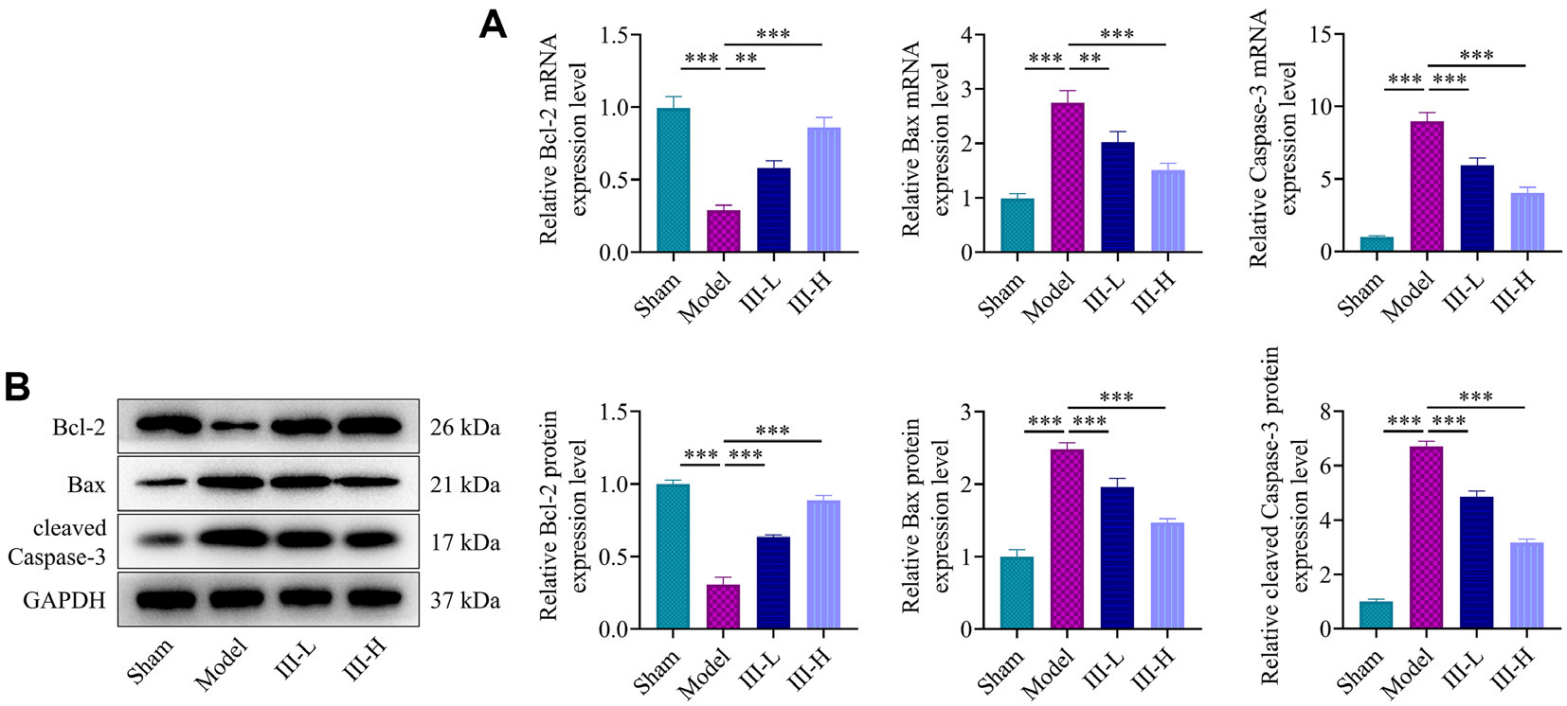
Regulation of apoptosis marker expressions by acute abdomen III in sepsis-induced rats. (**A**) Reverse transcription quantitative polymerase chain reaction (RT-qPCR) was conducted to evaluate mRNA levels of Bcl-2, Bax, and caspase-3 in intestinal tissue from sepsis-induced rats treated with or without acute abdomen III. GAPDH was used as a reference gene. (**B**) Western blot analysis of protein levels of Bcl-2, Bax, and cleaved Caspase-3 in sepsis-induced rats treated with or without acute abdomen III. GAPDH was used as a loading control. **P < 0.01; ***P < 0.001. Each group consisted of 3 rats.

To further explore the regulatory role of acute abdomen III in sepsis-induced intestinal cell apoptosis, expression levels related to apoptosis were examined. Bcl-2 (anti-apoptotic factor) was lowly expressed in the model group when compared to the sham group, whereas Bax (pro-apoptotic factor) and cleaved Caspase-3 were highly expressed, according to immunohistochemistry results (Fig. 4). In the model rats, acute abdomen III treatment waned Bax and cleaved Caspase-3 expressions while enhancing Bcl-2 expression (Fig. 4). Similar results were obtained in RT-qPCR, where higher mRNA levels of caspase-3 and Bax and lower Bcl-2 level were detected in the model group (*P* < 0.001, Fig. 5A). Bax and caspase-3 mRNA levels were lower but Bcl-2 mRNA level was higher in the acute abdomen III groups than in the model group (*P* < 0.01, Fig. 5A). Also, Western blot analysis supported these findings. In contrast to the sham group, Bcl-2 protein expression was decreased, whilst Bax and cleaved Caspase-3 levels were increased in the model group (*P* < 0.001, Fig. 5B). Acute abdomen III treatment elevated Bcl-2 protein expression while diminishing Bax and cleaved Caspase-3 protein expressions (*P* < 0.001, Fig. 5B).

## Discussion

Acute abdomen III, originating from a modified classic Chinese herbal formula, has been used in clinical practice for many years [14] and has assorted components with abundant properties. Acute abdomen III can improve the severity of patients with gastrointestinal function injury in sepsis, alleviate clinical symptoms in patients with severe pancreatitis, protect gastrointestinal function and reduce inflammation [17]. In addition, in rats with ventilator-associated pneumonia, acute abdomen III treatment can reduce the intestinal mucosal permeability, suppress bacterial translocation and protect the intestinal mucosal barrier [18]. These studies supported the clinical usability of acute abdomen III and its potential therapeutic effects on a variety of diseases. *Magnolia officinalis*, a key component of acute abdomen III, has been demonstrated in young rats to reduce recurrent status epilepticus-induced inflammation and damage [19] . Flavonoids from *Aurantii fructus*, another crucial ingredient, can alleviate disease through multi-targeted mechanisms including antioxidation, anti-inflammation, and anti-tumor effects [20]. *Salvia miltiorrhiza*, a well-known medicinal herb, holds significant therapeutic value. Zhang *et al*. found that components from *S. miltiorrhiza* exhibit neuroprotective potential against Alzheimer’s disease based on its antioxidation, anti-apoptosis, and anti-inflammation activities [21]. Furthermore, Wang *et al*. demonstrated that *Sargentodoxa cuneata* can mitigate epithelial barrier damage by blocking necroptotic signaling, and alleviate colitis [22]. Cha *et al*. proved that treatment with *Taraxaci herba* reduces blood concentrations of IL-6, IL-1β, and TNF-α, and improves symptoms of Behçet’s disease [23]. Our results indicated that acute abdomen III could lower blood concentrations of IL-6, IL-1β, and TNF-α in the sepsis model, consistent with previous findings. These outcomes evidenced that acute abdomen III can reduce systemic inflammation and associated symptoms.

Oxidative stress is a process involving cellular damage due to the excessive accumulation of free radicals and oxidizing substances [24]. It is a significant contributor to compromised intestinal barrier function, leading to increased permeability and bacterial translocation [25]. Liu *et al*. found that inhibiting Fgr kinase through the SIRT1/PGC-1α signaling pathway can alleviate oxidative stress, thus mitigating sepsis-related encephalopathy [26]. Melatonin relieves sepsis-induced intestinal injury through SIRT3-mediated reduction of oxidative stress [27]. Our study demonstrated that acute abdomen III suppressed oxidative stress in intestinal cells. Furthermore, elevated concentrations of endotoxin, D-lactate, and DAO have been detected in sepsis patients, indicating a compromised intestinal barrier [28]. High circulating levels of lactate correlate with the severity and mortality of sepsis [29]. Cao *et al*. reported that rapamycin ameliorates sepsis-induced intestinal barrier disruption by inhibiting DAO levels [30]. Herein, we found that acute abdomen III lowered the endotoxin, D-lactate, and DAO levels in sepsis, and also enhanced the expressions of tight junction proteins (ZO-1, claudin-1, and occludin), which were crucial in maintaining intestinal epithelial integrity. Disruption of these proteins, often mediated by pro-inflammatory cytokines like TNF-α, has been linked to increased intestinal permeability [31]. In summary, acute abdomen III reduced sepsis-induced inflammation, thereby promoting the levels of ZO-1, claudin-1 and occludin, which implied its role in protecting intestinal barrier function.

Apoptosis is instrumental in maintaining tissue homeostasis and clearing damaged cells [32]. However, excessive apoptosis in sepsis causes significant tissue damage and attenuates function [33], allowing bacteria and endotoxins to move from the intestinal lumen into bloodstream, and amplifying systemic inflammatory response [34]. Reducing intestinal epithelial cell apoptosis improves survival rates in sepsis mouse model [35]. The STING signaling pathway is implicated in promoting intestinal epithelial cell apoptosis, leading to increased intestinal permeability and bacterial translocation during sepsis [36]. Our findings indicated that acute abdomen III can modulate the levels of apoptosis-related proteins and inhibit intestinal epithelial cell apoptosis in sepsis model. Pro-inflammatory cytokines (such as TNF-α and IL-6) play essential roles in inducing intestinal epithelial cell apoptosis, and oxidative stress further exacerbates cell damage and death [37]. These factors collectively weakened the intestinal barrier, resulting in a vicious cycle of inflammation and organ dysfunction. Therefore, acute abdomen III may help maintain the intestinal barrier in sepsis rats by reducing inflammation and oxidative stress to inhibit intestinal epithelial cell apoptosis.

Of note, this study had several limitations to be addressed. First, there was a lack of investigation into possible side effects of acute abdomen III treatment. Second, while our study demonstrated that acute abdomen III can reduce sepsis-induced intestinal damage, the underlying mechanisms remained unclear. Further research is needed to fully understand how acute abdomen III interacts with inflammatory and oxidative stress pathways at the cellular and molecular levels.

In summary, our study demonstrates that acute abdomen III can mitigate sepsis-induced intestinal damage in a rat model through reducing systemic inflammation, decreasing oxidative stress, and improving intestinal barrier integrity. Acute abdomen III also promotes the expressions of tight junction proteins, while inhibiting apoptosis of intestinal epithelial cells. These results imply that acute abdomen III shields against sepsis-induced complications by maintaining intestinal barrier function and reducing inflammatory responses.
